# Predicting mortality in The Irish Longitudinal Study on Ageing (TILDA): development of a four-year index and comparison with international measures

**DOI:** 10.1186/s12877-022-03196-z

**Published:** 2022-06-21

**Authors:** Soraya Matthews, Mark Ward, Anne Nolan, Charles Normand, Rose Anne Kenny, Peter May

**Affiliations:** 1 Centre for Health Policy and Management, Trinity College Dublin, 3-4 Foster PlaceDublin 2, Dublin, Ireland; 2 The Irish Longitudinal Study On Ageing, Trinity College Dublin, Dublin, Ireland; 3 Economic and Social Research Institute (ESRI), Dublin, Ireland; 4 Cicely Saunders Institute Of Palliative Care, Policy & Rehabilitation, London, UK

**Keywords:** Older people, Risk factors, Mortality, Prognostication, Frailty

## Abstract

**Objectives:**

We aimed to replicate existing international (US and UK) mortality indices using Irish data. We developed and validated a four-year mortality index for adults aged 50 + in Ireland and compared performance with these international indices. We then extended this model by including additional predictors (self-report and healthcare utilization) and compared its performance to our replication model.

**Methods:**

Eight thousand one hundred seventy-four participants in The Irish Longitudinal Study on Ageing were split for development (*n* = 4,121) and validation (*n* = 4,053). Six baseline predictor categories were examined (67 variables total): demographics; cardiovascular-related illness; non-cardiovascular illness; health and lifestyle variables; functional variables; self-report (wellbeing and social connectedness) and healthcare utilization. We identified variables independently associated with four-year mortality in the development cohort and attached these variables a weight according to strength of association. We summed the weights to calculate a single index score for each participant and evaluated predicted accuracy in the validation cohort.

**Results:**

Our final 14-predictor (extended) model assigned risk points for: male (1pt); age (65–69: 2pts; 70–74: 4 pts; 75–79: 4pts; 80–84: 6pts; 85 + : 7pts); heart attack (1pt); cancer (3pts); smoked past age 30 (2pts); difficulty walking 100 m (2pts); difficulty using the toilet (3pts); difficulty lifting 10lbs (1pts); poor self-reported health (1pt); and hospital admission in previous year (1pt). Index discrimination was strong (ROC area = 0.78).

**Discussion:**

Our index is predictive of four-year mortality in community-dwelling older Irish adults. Comparisons with the international indices show that our 12-predictor (replication) model performed well and suggests that generalisability is high. Our 14-predictor (extended) model showed modest improvements compared to the 12-predictor model.

**Supplementary Information:**

The online version contains supplementary material available at 10.1186/s12877-022-03196-z.

## Introduction

### Background

Advances in medicine and improvements in public health have resulted in more people living longer with chronic illnesses for which there are few or no curative treatments [1–3]. Identifying individuals who have a higher mortality risk, especially earlier in their illness trajectory, can assist with reassessing goals of care, revaluating effective treatments, illness manageability, exploring psychosocial and physical issues, and initiating palliative care referral [4, 5]. Mortality prediction also has important applications in research and policy [6, 7].

Various prognostic indices to evaluate long-term mortality prediction exist [8, 9]. The quality of such studies is variable, often due to data limitations [10]; the strongest design is to utilise a large sample divided for derivation and validation, evaluated at baseline on a wide range of potentially relevant predictors, and followed reliably from baseline to death [11, 12]. The Gateway to Global Aging family of longitudinal ageing studies offer data with these characteristics in multiple countries [13]. In 2006 in the United States, Lee and colleagues developed a four-year mortality index for the general population of adults aged 50 + using data from the Health and Retirement Study (HRS) [14]. Kobayashi and colleagues (2017) followed this template using the HRS’s sister study English Longitudinal Study of Ageing (ELSA) to develop a ten-year mortality index for the general population of adults aged 50 + in England [15]. Both indices identified the age, sex, comorbid and functional characteristics associated with mortality, assigned each characteristic points based on the strength of that association, and derived the index as the sum of these points. A key strength of this approach is simplicity and parsimony, increasing usability by others. For example, calculating scale scores of functional limitations require six or eight specific data collection points. Such data collection is time-consuming in clinical practice and the data points may not be routinely available in data for research or policy. By identifying specific data points that can be elicited from routine data or very quick patient interactions (e.g. need assistance toileting), investigators have derived indices that are simple for others to apply. Both reported excellent discrimination and have been widely cited.

### Aim and rationale

This study aimed to develop and validate an index that predicts four-year mortality in the general population aged 50 + in the Republic of Ireland, using the HRS sister study The Irish Longitudinal Study on Ageing (TILDA). First, we used TILDA data to replicate methods from the mortality indices in HRS and ELSA. Second, we compared performance of the Irish, US and English indices in TILDA data to assess how important local data inputs are to predictive power. Third, we extended this approach by including additional healthcare utilization and self-report wellbeing and social connectedness variables using TILDA data. This third aim allows us to identify important additional variables that are easy to elicit in clinical practice, and/or recorded in medical files, that are expected to be associated with mortality. For example, prior hospitalisation is a well-known risk in other screening tools [16, 17]. Similarly, self-reported wellbeing and social connectedness have also been shown to have strong relationships with mortality risk in previous research [18–21]. As healthcare utilization and self-report wellbeing and social connectedness variables have not been employed in the HRS or ELSA mortality indices, we can assess if their inclusion improves prior prediction efforts. Our results from each of these aims can inform policy preparation and planning of future health service provision, and for epidemiological research.

## Methods

### Study design and setting

We conducted secondary analysis of longitudinal cohort data. We split the sample 50:50 into derivation and validation; we identified in the derivation sample those factors associated with four-year mortality; we weighted all statistically significant factors according to strength of association and summed these weights so that each TILDA participant had a total index score based on their characteristics at Wave 1. We assessed performance of the index in the validation sample.

Ireland is a country of approximately 4.98 million people in north-western Europe [22]. The Republic of Ireland refers to the 26 counties that are sovereign and separate to the additional six counties (Northern Ireland) that make up the island of Ireland. Compared to other countries in the EU, the Republic of Ireland has a relatively young population [23], meaning that the country will have proportionally larger numbers transitioning into older age and retirement within the next 20–30 years compared to the EU average [24].

### Participants and data sources

TILDA is a biennial prospective nationally representative study of older adults residing in the community in the Republic of Ireland. At Wave 1 in 2009–2010, a total of 8,174 people aged 50 + were enrolled. These 8,174 participants comprised the eligible sample for this study, with all of our predictors taken at their Wave 1 enrolment. Full details of the TILDA study design, participant selection and data collection are available elsewhere [25, 26].

All predictors examined in this study are taken from the computer-assisted personal interview (CAPI) conducted face-to-face in participants’ place of residence at Wave 1. Questions used in this study relate to participants’ demographics; cardiovascular-related illness; non-cardiovascular illness; health and lifestyle variables; functional variables; and healthcare utilization.

The outcome of interest was all-cause four-year mortality. All deaths in Ireland are recorded with the General Register Office (GRO), and TILDA data are linked to GRO data to March 2018, in a process detailed elsewhere [27]. Additionally, TILDA may learn of participant deaths after being contacted by a family member or after approaching for an interview. Therefore, for this study we have a mortality file providing full coverage of death dates within Ireland during the study period (via GRO) and additional non-comprehensive information on deaths outside the State (from family members).

### Variables and data measurement

We calculated the date 1,461 days (i.e. 365.25 × 4) after each individual’s Wave 1 CAPI was conducted and cross-referenced with the mortality file to give each participant a binary outcome for that date (= 1 if died within 1,461 days; = 0 if alive after 1,461 days). For this study to correspond to the HRS & ELSA mortality indices, the predictors of interest to evaluate mortality risk were analysed across six main categories [14, 15]. Table 1 shows the variables that we used following the Lee et al. (2006) approach: demographics; cardiovascular (CV)-related illness; non-CV diagnosis of serious illness; health and lifestyle variables; functional variables. Table 2 shows additional variables that we used to extend their approach: self-report wellbeing and social connectedness, and healthcare utilization.

**Table 1:** Descriptive statistics for the development and validation cohorts

		**Development (*N* = 4,121)**		**Validation (*N* = 4,053)**	
***Demographics***		***N***	**%**	***N***	**%**
Gender	*Male*	1,887	46	1,857	46
Age (Years)	*50–54*	803	19	819	20
	*55–59*	829	20	822	20
	*60–64*	717	31	677	29
	*65–69*	599	15	600	15
	*70–74*	475	12	490	12
	*75–79*	374	9	341	8
	*80–84*	213	5	183	5
	*85* +	110	3	121	3
***Cardiovascular (CV) Illness***					
Heart attack		186	5	192	5
Chronic heart failure		49	1	38	1
Stroke		61	1	72	2
Angina		233	6	216	5
Arrhythmia		578	14	534	13
High cholesterol		1,596	39	1,515	37
High blood pressure		1,561	38	1,470	36
***Non-CV Serious Illness***					
Diabetes		323	8	311	8
Cancer		230	6	282	7
Lung disease		175	4	155	4
Dementia		9	0	6	0
Psychological/Emotional problems		335	8	358	9
Arthritis		1,112	27	1,143	28
***Health Behaviour***					
Smoked past age 30		1,096	27	1,082	27
Smoke currently		749	18	741	18
Ever smoked		2312	56	2295	57
Drink alcohol daily		223	7	214	6
Often troubled by pain		1,466	36	1,430	35
Fallen in last year		808	20	775	19
Incontinence in last year		520	13	504	12
Visual impairment		689	17	684	17
Hearing impairment		88	2	106	3
***Functional Variables***					
*Activities of daily living difficulty*					
Bathing		129	3	142	4
Using the toilet		38	1	53	1
Eating		24	1	34	1
Walking across a room		43	1	54	1
Dressing		245	6	271	7
Getting out of bed		74	2	76	2
*Instrumental Activities of daily living difficulty*					
Using the phone		26	1	32	1
Shopping for groceries		147	4	170	4
Preparing meals		83	2	109	3
Taking medication		34	1	51	1
Managing money		50	1	77	2
Doing household chores		185	4	211	5
*Other measures of functional difficulties*					
Walking 100 m		303	7	298	7
Climbing 1 flight of stairs		302	7	331	8
Climbing several flights of stairs		1,245	30	1,250	31
Pushing/pulling heavy objects		534	13	507	13
Getting out of bed		74	2	76	2
Vigorous physical exercise in last week		1,046	25	1,026	25
Lifting 10lbs		786	19	710	18
Stooping, crouching, kneeling		1,129	27	1,133	28
Reach above shoulders		304	7	347	9
Picking up a coin		151	4	180	4
Getting up from a chair		698	17	772	19
Sitting for long periods of time		425	10	461	11

**Table 2:** Descriptive statistics for the additional self-report variables

	**Development (*N* = 4,121)**		**Validation (*N* = 4,053)**	
	***N***	**%**	***N***	**%**
***Self-Report Wellbeing***				
**Described physical health as ‘poor’**	198	5	219	5
** Described mental health as ‘poor’**	53	1	66	2
** Reported having a long-term health problem, illness, disability or infirmity**	1,556	38	1,575	39
** Sleep is restless all the time**	288	7	314	8
** Difficulty falling asleep all the time**	423	10	459	11
** Feel depressed all the time**	55	1	58	1
***Self-Report Social Connectedness***				
** Feel lonely all the time**	89	2	85	2
** Participates in sports/social groups**	1,978	48	1,917	47
** Has zero relatives they feel close to**	486	12	495	12
** Has zero friends they feel close to**	461	11	404	10
***Healthcare Utilisation***				
** More than 50% chance of moving into nursing home in next 5yrs**	143	4	144	4
** More than 50% chance of developing cognitive problems aged 75 + **	2,778	92	2,711	92
** More than 1 ED or inpatient admission in last year**	835	20	885	22

### Bias, missing data and loss to follow-up

With respect to external validity, bias concerns are very low. TILDA Wave 1 was sampled in a sophisticated way to represent the population of interest [28]. With respect to internal validity, the biggest concern was missing values, which can arise if a participant refused to respond to a question or did not know the relevant answer.

In relation to missingness, preliminary checks showed that our included predictors demonstrated either zero missingness or very low missingness (< 0.75%) in the total sample. There were two exceptions to this: drinking alcohol daily (16.9%) and BMI (28.2%). However, both of these variables were excluded after the initial bivariate analyses, as per Step one of our statistical methods (Supplementary Material), due to lack of statistical significance with the outcome. We tested the robustness of our main results to different imputation methods where data were missing. We first dropped the missing subjects and second imputed age-adjusted median values. Results did not substantively change. Loss to follow-up should be low because we have full GRO coverage, but it is possible that we missed some deaths that occurred outside Ireland and that have not become apparent in later waves when we tried to conduct an interview.

### Statistical Methods

We followed and then extended the template already established to develop mortality indices in HRS [14] and ELSA [15]. Our statistical methods encompassed 10 steps and are detailed fully in the Supplementary Material. Briefly, we randomly divided the 8,174 participants into development and validation cohorts. In the development sample we checked association between each Table 1 predictor and outcome, first in bivariate regressions, then in multivariable regressions, using Bayesian Information Criteria (BIC) to refine the model. We tested the final model in order to check stability to different approaches to variable selection and then allocated each predictor a number of points where the smallest coefficient was worth one point and all other coefficients were scaled accordingly. In both the development and validation samples, discrimination of the model was evaluated using the area under the receiver operating characteristic (ROC) curves in both cohorts. We then examined the effect of including Table 2 variables as additional predictors in our mortality index, following the same steps. Finally, we compared the performance of our index to that those of Lee et al. (2006) [14] and Kobayashi et al. (2017) [15] using the chi-square test of equality of ROC areas. All statistical analyses were conducted using StataSE 16.1 [29].

### Sensitivity analyses

For sensitivity analysis we established two and six-year mortality outcome variables for comparison. We checked sensitivity of results to missing data using different imputation strategies.

## Results

### Characteristics of the sample

In Wave 1 (2009–2010) of TILDA, 8,174 individuals aged 50 + completed data collection, and we divided these randomly into development (*n* = 4,121) and validation (*n* = 4,053) cohorts.

A total of 448 (5.5%) participants died within four years of enrolment, 217 participants in the development cohort (5.2%) and 231 participants in the validation cohort (5.7%). Descriptive characteristics of both cohorts were comparable and can be found in Tables 1 and 2.

### Index Development

Following Lee et al. (2006), we first regressed every variable in Table 1 on four-year mortality in bivariate regressions [14]. Associations are shown in Table 3; each predictor is binary so each association represents the estimated odds that a participant died within four years of enrolment if having a value of 1 for the predictor versus having a value of 0 for the predictor. Of the 52 predictors, 42 were significantly associated with mortality risk in bivariate regression.

**Table 3:** Bivariate analysis of risk factors and 4-year mortality in development cohort

		**No. of Deaths for each predictor N (%)**		**Odds Ratio**	**95% Confidence Interval**
***Demographics***					
Gender	*Female*	102 (47)		1.0	–
	*Male*	115 (53)		1.4	(1.0–1.8)
Age (Years)	*50–54*	6 (3)		0.1	(0.05–0.3)
	*55–59*	18 (8)		0.3	(0.2–0.6)
	*60–64*	14 (6)		0.3	(0.2–0.5)
	*65–69*	25 (12)		0.8	(0.5–1.2)
	*70–74*	41 (19)		1.9	(1.3–2.7)
	*75–79*	44 (20)		2.8	(1.9–3.9)
	*80–84*	34 (16)		3.9	(2.6–5.7)
	*85* +	35 (16)		9.8	(6.4–15.1)
***CV diagnoses***		**Present**	**Absent**		
Heart attack		29 (13)	187 (86)	3.7	(2.4–5.6)
Chronic heart failure		7 (3)	209 (96)	3.1	(1.4–6.9)
Stroke		8 (4)	208 (96)	2.8	(1.3–5.9)
Angina		32 (15)	184 (85)	3.2	(2.1–4.8)
Arrythmia		49 (23)	167 (77)	1.9	(1.3–2.6)
High cholesterol		70 (32)	146 (67)	0.7	(0.6–1.0)
High blood pressure		104 (48)	112 (52)	1.6	(1.2–2.1)
***Non-CV diagnoses***		**Present**	**Absent**		
Diabetes		32 (15)	184 (85)	2.2	(1.5–3.2)
Cancer		36 (17)	181 (83)	3.8	(2.6–5.6)
Lung disease		24 (11)	193 (89)	3.1	(2.0–4.9)
Dementia		4 (2)	213 (98)	14.6	(3.9–54.9)
Psychological/Emotional problems		13 (6)	204 (94)	0.7	(0.4–1.3)
Arthritis		77 (35)	140 (65)	1.5	(1.1–2.0)
***Health Behaviour***		**Yes**	**No**		
Smoked past age 30		99 (46)	117 (54)	1.7	(1.2–2.3)
Smoke currently		58 (27)	159 (73)	2.5	(1.9–3.2)
Ever smoked		143 (66)	74 (34)	1.5	(1.2–2.1)
Often troubled by pain		82 (38)	135 (62)	1	(0.8–1.5)
Drink alcohol daily		11 (5)	147 (68)	1.1	(0.6–2.0)
Fallen in last year		58 (27)	159 (73)	1.5	(1.1–2.1)
Incontinence in last year		41 (19)	172 (79)	1.7	(1.2–2.4)
Visual impairment		85 (39)	132 (61)	3.5	(2.6–4.7)
Hearing impairment		7 (3)	210 (97)	1.6	(0.7–3.5)
***Functional—‘Difficulty with’***		**Yes**	**No**		
Bathing		26 (12)	191 (88)	5.0	(3.2–7.9)
Using the toilet		13 (6)	204 (94)	9.9	(5.0–19.6)
Eating		8 (4)	209 (96)	9.3	(3.9–22.0)
Walking across a room		11 (5)	206 (95)	6.5	(3.2–13.0)
Dressing		29 (13)	188 (87)	2.6	(1.7–4.0)
Getting out of bed		18 (8)	199 (92)	6.2	(3.6–10.8)
Using the phone		7 (3)	210 (97)	6.8	(2.8–16.4)
Shopping for groceries		35 (16)	182 (84)	6.5	(4.3–9.8)
Preparing meals		29 (13)	188 (87)	11.0	(6.8–17.7)
Taking medication		11 (5)	206 (95)	9.0	(4.3–18.7)
Managing money		11 (5)	206 (95)	5.3	(2.7–10.5)
Doing household chores		40 (18)	177 (82)	5.9	(4.0–8.6)
Walking 100 m		65 (30)	152 (70)	6.6	(4.8–9.1)
Climbing 1 flight of stairs		59 (27)	158 (73)	5.6	(4.1–7.8)
Climbing several flights of stairs		133 (61)	84 (39)	4.0	(3.0–5.3)
Pushing/pulling heavy objects		67 (31)	150 (69)	3.3	(2.4–4.5)
Vigorous physical exercise in last week		21 (10)	196 (90)	0.3	(0.2–0.5)
Lifting 10lbs		95 (44)	122 (56)	3.6	(2.7–4.8)
Stooping, crouching, kneeling		105 (48)	112 (52)	2.6	(2.0–3.5)
Reach above shoulders		33 (15)	184 (85)	2.4	(1.6–3.6)
Picking up a coin		21 (10)	196 (90)	3.1	(1.9–5.0)
Getting up from a chair		61 (28)	156 (72)	2.0	(1.5–2.7)
Sitting for long periods of time		33 (15)	184 (85)	1.6	(1.1–2.4)

Due to missing data, the sum of deaths for each variable may vary

We entered these predictors into a multivariable regression using stepwise backward selection and the model retained 20 significant predictors of mortality. Following stability checks forwards and backwards, testing the stability of the list of predictors on statistical significance and BIC, our model using the Lee/Kobayashi approach included gender, age; diagnoses of heart attack and cancer; being a smoker past age 30; and difficulty walking 100 m, using the toilet and lifting 10lbs (Table 4).

**Table 4:** Independent risk factors for 4-year mortality in the development cohort (*N* = 4,121) in the multivariable analysis

Risk Factor	Adjusted OR (95% CI)* 12 Predictor Model	Points	Adjusted OR (95% CI)* 14 Predictor Model	Points
***Demographics***				
**Male**	1.5 (1.1–2.1)	1	1.5 (1.1–2.1)	1
**Age 65–69**	2.5 (1.5–4.2)	2	2.5 (1.5–4.2)	2
**Age 70–74**	4.6 (2.9–7.4)	4	4.7 (2.9–7.6)	4
**Age 75–79**	5.9 (3.6–9.5)	4	6.0 (3.7–9.8)	4
**Age 80–84**	10.7 (6.4–17.9)	5	11.2 (6.7–18.8)	6
**Age 85 + **	20.6 (11.9–35.7)	7	23.9 (13.7–41.6)	7
***Cardiovascular (CV) Illness***				
**Heart Attack**	1.9 (1.2–3.1)	1	1.8 (1.1–2.9)	1
***Non-CV diagnosis of serious illness***				
**Cancer**	3.5 (2.3–5.5)	3	3.3 (2.1–5.1)	3
***Other Health & Lifestyle Factors***				
**Smoked past age 30**	2.2 (1.6–3.6)	2	2.2 (1.6–2.9)	2
***Functional Variables—‘Difficulty with’***				
*Activities of daily living*				
**Walk 100 m**	2.4 (1.6–3.5)	2	2.1 (1.4–3.1)	2
**Using the toilet**	3.5 (1.5–8.1)	3	3.2 (1.4–5.4)	3
*Other measures of functional difficulties*				
**Lifting 10lbs**	1.8 (1.3–2.5)	1	1.6 (1.1–2.3)	1
***Self-Report Wellbeing***				
**Described physical health as ‘poor’**			1.7 (1.1–2.8)	1
***Healthcare Utilization***				
**More than 1 ED or inpatient admission in last year**			1.7 (1.2–2.4)	1
***ROC***				
**Development cohort**	**0.816**		**0.825**	
**Validation cohort**	**0.774**		**0.783**	

*CI* confidence interval, *OR* odds ratio. Each OR was adjusted for the other risk factors in the table. *For age, the reference group is those aged 50–64

We then checked whether this index was further improved by the inclusion of self-report wellbeing and social connectedness variables, and health care utilization predictors that are recorded in the TILDA survey and may be hypothesised as associated with mortality (Table 2). We performed a multivariable regression with the predictors identified using the Lee/Kobayashi approach and each additional predictor in turn, assessing model performance on BIC. Two predictors improved BIC performance: self-reported physical health described as poor, and hospital admission (ED or inpatient) in the last 12 months. When both variables were added together in a 14-variable model, this further improved BIC and all variables were significant at p < 0.05. The 14-predictor model improved discriminatory power in development and validation cohorts (ROC = 0.825 and ROC = 0.783, respectively), compared to the 12-predictor model (ROC = 0.816 and ROC = 0.774).

This model, presented in Table 4, is our final four-year mortality index for the Republic of Ireland. A risk score is calculated for each participant by adding the points for each risk factor present using the point system in Table 4. Within the 14-predictor model, the lowest score available is 0 points and highest is 22 points. A 65-year-old (2 points) male (1 point) who smoked past age 30 (2 points), has cancer (3 points) and has difficulty walking 100 m (1 point), but no other diagnoses or problems listed, would have a risk score of nine points (Table 4).

### Model performance, risk stratification and international comparisons

For accurate comparisons, we evaluated the original 12-predictor model within the development and validation cohorts, and used that model to compare performances to that of the Lee et al. (2006) [14] and Kobayashi et al. (2017) [15] indices (Table 5). The point-based model showed excellent discrimination, with a ROC area of 0.816 in the development cohort and 0.774 in the validation cohort. Our model also showed great discrimination compared to the Lee et al. (2006) [14] and Kobayashi et al. (2017) [15] indices (0.784 and 0.779, respectively). Mortality rates ranged from < 1% (3/741; development cohort), 1% (5/720; validation cohort), < 1% (0/372; Lee et al., 2006) and 1% (2/393; Kobayashi et al., 2017) at the 0 point level to 75% (3/4; development cohort), 43% (3/7; validation cohort), 35% (8/23; Lee et al., 2006), and 9% (8/94; Kobayashi et al., 2017) at the ≥ 14 point level.

**Table 5:** Validation of the prognostic index: comparing the 12-predictor model performance by point score and comparing our index to the performances of the Lee et al. (2006) & Kobayashi et al. (2017) indices

	**No. of individuals who died/ No. of individuals at risk (%)**			
Point Score	**Development Cohort**	**Validation Cohort**	**Lee et al. (2006)**	**Kobayashi et al. (2017)**
0	3/741 (< 1)	5/720 (1)	0/372 (< 1)	2/393 (1)
1	6/815 (1)	9/786 (1)	5/379 (1)	4/439 (1)
2	7/427 (2)	10/445 (2)	10/785 (1)	9/523 (2)
3	11/554 (2)	21/548 (4)	14/610 (2)	18/676 (3)
4	13/378 (3)	13/337 (4)	26/542 (5)	8/306 (3)
5	30/429 (7)	31/466 (7)	17/436 (4)	16/418 (4)
6	23/222 (10)	28/211 (13)	21/307 (7)	8/213 (4)
7	33/219 (15)	22/184 (12)	38/241 (16)	26/373 (7)
8	25/131 (19)	29/147 (20)	18/122 (15)	12/170 (7)
9	18/75 (24)	19/78 (24)	17/91 (19)	35/209 (17)
10	20/64 (31)	24/62 (39)	20/58 (34)	13/92 (14)
11	10/24 (42)	9/29 (31)	10/32 (31)	17/64 (27)
12	6/11 (56)	5/9 (56)	18/40 (45)	11/51 (22)
13	8/14 (57)	2/15 (13)	6/15 (40)	10/32 (31)
> 14	3/4 (75)	3/7 (43)	8/23 (35)	8/94 (9)
ROC Area	0.816	0.774	0.784	0.779

*ROC* receiver operating characteristic

### Risk stratification by points and age groups

**Fig. 1:**
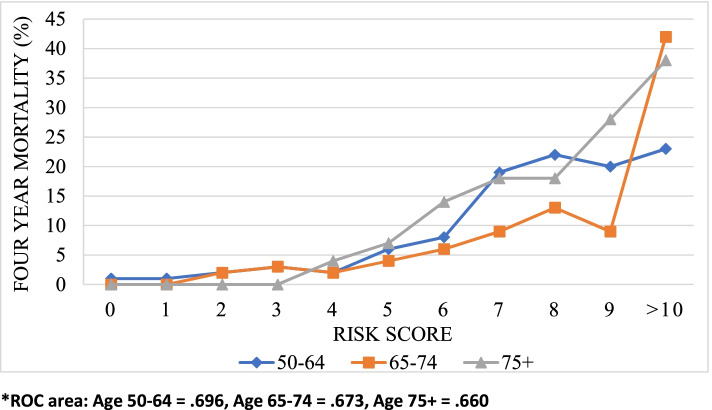
14-Predictor model of four-year mortality by risk score in differing age groups. Note. 50–64 (N = 2,349), 65–74 = (N = 1,074) & 75 + (N = 697) [See additional file 1]

We evaluated the point scores for three different age groups using the 14-predictor model and found that discrimination power was satisfactory within each group within the development cohort (Fig. 1). The ROC area for age 50–64 was 0.696, age 65–74 was 0.673, and age 75 + was 0.660. We interpreted these results as indicating the 14-predictor index has similar predictive power across the age distribution of our sample.

## Discussion

### Key results

We developed and validated a predictive index for four-year mortality in Ireland using high-quality longitudinal survey data of community-dwelling people aged over 50. Our aims of replicating and extending prior mortality indices were achieved. The 12-predictor replication model showed good discriminatory power within our development and validation cohorts, and performed well when compared to with the US[14] and UK[15] indices. The final 14-predictor extended model showed only small improvements in discriminatory power within development and validation cohorts (Table 4), and similar accuracy across different age groups (Fig. 1). Our approach followed closely prior mortality prediction efforts in the US [14] and England [15]. Our index structure exhibited a high level of consistency with those, incorporating age, gender, diagnoses of disease and functional limitations. Functional limitations data are critical to the success of these indices because they capture aspects of the severity of disease, supplementing the information in a binary diagnosis variable and thus improving on the predictive power of comorbidity indices. We extended the approach of those prior indices to include additional self-report wellbeing and social connectedness variables and healthcare utilization variables, but these resulted in only a modest improvement in model performance. While multiple studies have shown that health care use and self-reported health are significantly associated with mortality, they do not in our data substantively improve efforts to address the prediction problem. As such our results are consistent with literature elsewhere: improving the specificity of prediction tools likely requires data points that are not routinely collected [30].

Comparing the composition of different indices showed differences with both the US (where BMI, diabetes and lung disease were all risk factors) and with England (where lung disease and lack of physical activity were risk factors). There were also differences in the specific functional limitations that ended up in each model. However, when we evaluated the HRS and ELSA indices in our validation sample, all three indices had very similar performance (Table 5). International generalisability appears high, and the relevance of specific functional limitations appears low provided functional capacity is captured.

Our index may have applications in care, research and policy. In clinical settings, it is a simple guide to discriminating between individuals at low and high risk of mortality. An individual’s prognosis may be important for targeting treatments, particularly those with a long benefits horizon, e.g. cancer screening; for lifestyle advice; and for prompting goals-of-care discussions and advance care directives [9, 31]. In research, prognosis may be an important factor in designing trials and sampling in observational studies [7]. It is also a potentially powerful predictor of outcomes. For example, it is well-established that proximity to death drives health care use but this is seldom controlled for prospectively [32]. In policy, quantifying mortality risk is critical to accurate estimation of clinical benefits and health care costs. For example, efforts to estimate the population-level health effects of the Coronavirus-19 pandemic require detailed risk stratification that goes beyond age and gender adjustment to capture mortality and morbidity [33].

### Limitations

Four-year mortality from TILDA baseline was 5.5%, compared to 12% in the HRS study for the same period and 25% for the ten-year mortality outcome studied in ELSA. Given the comprehensive linkage with death data in Ireland in the study period, this difference is most plausibly explained by the TILDA sample being younger and healthier at recruitment; the age profile of the studies reflects Ireland’s young population compared to other high-income countries [23]. This also meant that we had a much smaller number of cases on which to calculate the index. Smaller cell sizes increase the risk of uncertainty of the derived weights, which may harm generalisability. By splitting our sample into derivation and validation and comparing index performance in each, we show that internal validity is strong. By comparing performance of equivalent indices in other countries we show that external validity is strong. While it is possible that more cases in the data would change the weights and so improve the index performance, based on these internal and external comparisons there is no a priori reason to anticipate potential for large additional improvements. TILDA, like HRS, originally sampled community-dwelling adults, so the index will not be applicable in residential care populations. Future maturity of the TILDA sample, and updated GRO linkage, will allow us to investigate questions of sampling and sample size. For example, the timing of disease diagnosis, and the trajectories of lifestyle factors and functional difficulties, will likely be predictive of mortality. As the TILDA study adds more waves, and so investigators can employ some waves prior to baseline and have sufficient follow-up data, such analyses are planned. All included predictors are collected through self-report and interviews and may be exposed to recall error or bias. However, interviewer assistance, use of CAPI and data analysis methods are designed to combat this.

## Conclusion

Our model comparisons with the US [14] and UK [15] indices shows that our 12-predictor (original replication) model performed well, and this replication suggests that generalisability is high across countries. Our 14-predictor (extended) model showed modest improvements compared to the 12-predictor model, indicating that their statistical utility is similar.

Our final 14-variable index offers a potentially useful tool that can predict four-year mortality in older community-dwelling adults in the Republic of Ireland. It can be delivered during patient interactions without the need for a full clinical history and utilised to develop care strategies. It can also serve as an instrument for future epidemiological research and policy and be used as a comparator tool for international populations.

## Supplementary Information

**Additional file 1.** Statistical Methods and Variables included in Analysis.

## Data Availability

TILDA data can be accessed in two ways. Access to all data, including the mortality file, is available only from the TILDA servers at Trinity College Dublin. Application for access can be made via their website: https://tilda.tcd.ie/data/accessing-data/. Access to a reduced, harmonized version of Waves 1–4 can be accessed on application here: https://www.ucd.ie/issda/data/tilda/. TILDA recognizes replicability as an important part of science and on application can make available all.do files and data used in this study.

## Abbreviations

TILDA: The Irish Longitudinal Study on Ageing
HRS: Health and Retirement Study
ELSA: English Longitudinal Study of Ageing
CAPI: Computer-assisted personal interview
EU: European Union
GRO: General Register’s Office
CV: Cardiovascular
BIC: Bayesian Information Criteria
ED: Emergency Department
US: United States
UK: United Kingdom
ROC: Receiver operating characteristic

## References

[1] Dall TM, Gallo PD, Chakrabarti R, West T, Semilla AP, Storm MV (2013). An aging population and growing disease burden will require a large and specialized health care workforce by 2025. Health Aff (Millwood).

[2] McPake B, Mahal A (2017). Addressing the Needs of an Aging Population in the Health System: The Australian Case. Health Syst Reform.

[3] Sleeman KE, de Brito M, Etkind S, Nkhoma K, Guo P, Higginson IJ (2019). The escalating global burden of serious health-related suffering: projections to 2060 by world regions, age groups, and health conditions. Lancet Glob Health.

[4] Avati A, Jung K, Harman S, Downing L, Ng A, Shah NH (2018). Improving palliative care with deep learning. BMC Med Inform Decis Mak.

[5] Lynn J (2001). Perspectives on care at the close of life. Serving patients who may die soon and their families: the role of hospice and other services. Jama.

[6] Kelly M, O'Brien K, Hannigan A. Using linked administrative health data for palliative and end of life care research in Ireland: potential and challenges. HRB Open Res; 2021. 10.12688/hrbopenres.13215.1 PMC8014706 33842831

[7] Riley RD, Moons KGM, Snell KIE, Ensor J, Hooft L, Altman DG (2019). A guide to systematic review and meta-analysis of prognostic factor studies. BMJ.

[8] Bloom CI, Ricciardi F, Smeeth L, Stone P, Quint JK (2019). Predicting COPD 1-year mortality using prognostic predictors routinely measured in primary care. BMC Med.

[9] Yourman LC, Lee SJ, Schonberg MA, Widera EW, Smith AK (2012). Prognostic indices for older adults: a systematic review. JAMA.

[10] Kent P, Cancelliere C, Boyle E, Cassidy JD, Kongsted A (2020). A conceptual framework for prognostic research. BMC Med Res Methodol.

[11] Riley RD, Hayden JA, Steyerberg EW, Moons KG, Abrams K, Kyzas PA (2013). Prognosis Research Strategy (PROGRESS) 2: prognostic factor research. PLoS Med.

[12] Smith LJ-E, Moore E, Ali I, Smeeth L, Stone P, Quint JK (2017). Prognostic variables and scores identifying the end of life in COPD: a systematic review. Int J Chron Obstruct Pulmon Dis.

[13] Gateway to Global Aging Data. A platform for population survey data on aging around the world . 2021. Available from: https://g2aging.org/. Cited 06/06/2021.

[14] Lee SJ, Lindquist K, Segal MR, Covinsky KE (2006). Development and Validation of a Prognostic Index for 4-Year Mortality in Older Adults. JAMA.

[15] Kobayashi LC, Jackson SE, Lee SJ, Wardle J, Steptoe A (2017). The development and validation of an index to predict 10-year mortality risk in a longitudinal cohort of older English adults. Age Ageing.

[16] van Walraven C, Forster AJ (2017). The HOMR-Now! Model Accurately Predicts 1-Year Death Risk for Hospitalized Patients on Admission. Am J Med.

[17] Quinn KL, Stall NM, Yao Z, Stukel TA, Cram P, Detsky AS (2019). The risk of death within 5 years of first hospital admission in older adults. CMAJ.

[18] Benjamins MR, Hummer RA, Eberstein IW, Nam CB (2004). Self-reported health and adult mortality risk: an analysis of cause-specific mortality. Soc Sci Med.

[19] Wiest M, Schüz B, Webster N, Wurm S (2011). Subjective well-being and mortality revisited: Differential effects of cognitive and emotional facets of well-being on mortality. Health Psychol.

[20] Shor E, Roelfs DJ, Yogev T (2013). The strength of family ties: A meta-analysis and meta-regression of self-reported social support and mortality. Social Networks.

[21] Lorem G, Cook S, Leon DA, Emaus N, Schirmer H (2020). Self-reported health as a predictor of mortality: A cohort study of its relation to other health measurements and observation time. Sci Rep.

[22] Central Statistics Office. Population and Migration Estimates April 2020 [press release]. Online: Central Statistics Office; 2020. Available from: https://www.cso.ie/en/csolatestnews/pressreleases/2020pressreleases/pressstatementpopulationandmigrationestimatesapril2020/. Cited 01/06/2021.

[23] May P, Johnston BM, Normand C, Higginson IJ, Kenny RA, Ryan K (2019). Population-based palliative care planning in Ireland: how many people will live and die with serious illness to 2046?. HRB Open Res.

[24] Kane PM, Daveson BA, Ryan K, McQuillan R, Higginson IJ, Murtagh FE (2015). The need for palliative care in Ireland: a population-based estimate of palliative care using routine mortality data, inclusive of nonmalignant conditions. J Pain Symptom Manage.

[25] Donoghue OA, McGarrigle CA, Foley M, Fagan A, Meaney J, Kenny RA. Cohort Profile Update: The Irish Longitudinal Study on Ageing (TILDA). Int J Epidemiol. 2018;47(5):1398–l. 10.1093/ije/dyy163 30124849

[26] Kearney PM, Cronin H, O'Regan C, Kamiya Y, Savva GM, Whelan B (2011). Cohort profile: the Irish Longitudinal Study on Ageing. Int J Epidemiol.

[27] Ward M, May P, Briggs R, McNicholas T, Normand C, Kenny R (2020). Linking death registration and survey data: Procedures and cohort profile for The Irish Longitudinal Study on Ageing (TILDA) [version 2; peer review: 3 approved]. HRB Open Research..

[28] TILDA. The Irish Longitudinal study on Ageing (TILDA) Wave 1, 2009-2011. [dataset]. Version 1.9. Irish Social Science Data Archive. 2019; SN:0053-01. Available from: https://www.ucd.ie/issda/data/tilda/wave1.

[29] Stata Statistical Software: Release 16. 2019 [cited 08/03/2021]. Available from: https://www.stata.com/.

[30] Kelley AS, Bollens-Lund E. Identifying the Population with Serious Illness: The “Denominator” Challenge. J Palliative Med. 2018;21(S2):S-7-S-16. 10.1089/jpm.2017.0548 PMC5756466 29125784

[31] Hjelmfors L, van der Wal MH, Friedrichsen MJ, Mårtensson J, Strömberg A, Jaarsma T (2015). Patient-Nurse Communication about Prognosis and End-of-Life Care. J Palliat Med.

[32] Breyer F, Lorenz N (2021). The, “red herring” after 20 years: ageing and health care expenditures. Eur J Health Econ..

[33] Briggs AH, Goldstein DA, Kirwin E, Meacock R, Pandya A, Vanness DJ (2021). Estimating (quality-adjusted) life-year losses associated with deaths: With application to COVID-19. Health Econ.

