# Supplementary material for: Predicting mortality in The Irish Longitudinal Study on Ageing (TILDA): development of a four-year index and comparison with international measures
Source: BMC Geriatr. 2022 Jun 21;22:510. doi: 10.1186/s12877-022-03196-z (PMC9211047; doi:10.1186/s12877-022-03196-z)
Supplement: Supplementary file 1 — Additional file 1. Statistical Methods and Variables included in Analysis. [file 12877_2022_3196_MOESM1_ESM.docx]

**Predicting mortality in The Irish Longitudinal Study on Ageing (TILDA): Development of a four-year index and comparison with international measures**

**Page of Contents**

**Additional file 1**

1. Statistical Methods
2. Variables included in Analysis
3. **Statistical Methods**

This study followed the statistical guidance that was used to develop the published mortality indices based on the HRS (Lee, et al., 2006) and ELSA (Kobayashi et al., 2017) data. Our statistical methods encompassed 10 steps. As mentioned in previous sections, the development and validation cohorts were formulated, and mortality data were obtained for all decedents. Step one focused on the development cohort and analysing the bivariate relationships between each of the original 52 variables and 4-year mortality. Step two retained the 42 variables from step one that had *p*<0.05 and applied backward elimination (whereby *p* must continue to be <0.05 to retain). To achieve this, we ran a single multivariable regression and removed variables based on the highest *p* values (starting with *p>*0.9). We continued this process in rounds until all variables in the multivariable regression were *p*<0.05 (e.g. the next round removed *p*>0.8, then *p*>0.7, and so forth). The final model in step two contained 20 variables for mortality.

Step three aimed to simplify the model further by applying the Schwarz Bayesian Information Criterion (BIC) on the remining variables. This method will minimize losses in predictive ability. We ran the final model from step two in a multivariable regression and applied BIC to get a BIC score for baseline. We then repeatedly ran the final model omitting one variable each time to see if the BIC score had changed from the baseline. Any omitted variables that increased the BIC score compared to baseline needed to be addressed. The omitted variable that demonstrated the largest increase in BIC score was removed from the model and the next round would begin. The new model (minus the removed variable) was ran and BIC was applied to get the new baseline score. The process began again of omitting one variable each time to see if the BIC score changed and the variable with the largest increase was then removed. These rounds continued until all variables in the model demonstrated an decrease in BIC score from the baseline. The final model in step three retained 10 variables.

Step four focused on running stability checks on the final model from step three. The final model from step 3 was ran as a multivariable regression and BIC was applied for a baseline BIC score. Then the final model was ran with one extra variable (total of 11 variables) repeatedly and BIC was applied to see changes from BIC baseline. Each regression had a different variable substituted in as the extra variable. Each of the variables from step one (apart from grouped heart conditions and excess smoking variables) were individually added to the model to see if any of them had a negative effect on BIC score. Five of the additional variables reduced the BIC score. Two of these five variables had high collinearity. To address this, we dropped the variable that produced the higher BIC score. The next round in step four addressed the additional four variables by running six unique pairs of variables alongside the original model. For example, the original model would be ran with the addition of two out of the four extra variables. This would be re-ran until all combinations had been applied. The next round focused on running the same additional variables as four unique trebles alongside the original model. This would be re-ran until all combinations had been applied. The final round ran the original model and all four variables in one multivariable regression. The findings from all rounds showed that only one of the additional four variables contributed to the original model.

Carrying on from step four, further stability checks were applied in step five. In step one through three all variables were added to the model together regardless of grouping. For the next stability check, variables were grouped into the following categories: demographics; cardiovascular illness; non-cardiovascular diagnosis of serious illness; health and lifestyle factors; and functional variables. Multivariable regressions were ran for each grouping individually with four-year mortality. Any variables that were not *p*<0.05 were subsequently dropped. The remaining 26 variables were merged into one model. This model was ran again as a multivariable regression and any variables that were not *p*<0.05 were dropped. The model was ran repeatedly whilst dropping non-significant variables each time until all retained variables were *p*<0.05. The final model of step five had 14 variables.

Step six focused on applying the same method as step three (BIC) on the stability check model from step five. We ran the final model from step five in a multivariable regression and applied BIC to get a BIC score for baseline. We then repeatedly ran the final model omitting one variable each time to see if the BIC score had changed from the baseline. The omitted variable that demonstrated the largest increase in BIC score was removed from the model and the next round would begin. At the end of these rounds, 11 variables were retained in the final model.

Step seven focused on assessing the similarities and differences between the original model and the model after stability checks. Steps four to six applied stability checks as per the statistical guidance from Lee et al. (2017). Whereas the previous authors reported finding no differences between their original model and the model derived from stability checks, we did encounter differences between the models. We found that eight variables were consistent between the original and stability model. However, five variables were inconsistent (two variables from original model and three from stability model). To address this, we first ran both models separately and applied BIC. The original model demonstrated a lower BIC score than the stability model, so we chose the original model to use as our baseline model. Firstly, we ran a multivariable regression and BIC with the original model and added one of the three inconsistent variables from the stability model. This was repeated for all three variables. Secondly, we ran three unique pairs of variables alongside the original model. For example, the original model would be ran with the addition of two out of the three inconsistent variables. This would be re-ran until all combinations had been applied. Finally, the original model was ran with all three of the inconsistent variables included and BIC was applied. It was found that all three of the variables decreased BIC scores, however based on *p* values, one of the variables was dropped.

Step eight aimed to validate the model against the previously assigned validation cohort. Both the original model from step three and the updated stability model from step seven were tested. The original model contained 10 variables and each of them were assigned a weight in points based on their coefficients. This was calculated by using the variable with the lowest coefficient as the baseline weight. The same weight calculations were applied to the stability check model (12 variables). To validate each model, we calculated the receiver operating characteristic (ROC) curves for each model using the validation cohort. Once we had a ROC *p* value score for each model (original & stability), we then compared the scores to the ROC *p* values for the Lee et al. (2006) and Kobayashi et al. (2017) cohorts. It was found that the stability model from step seven demonstrated a better ROC *p* value, and was more in line with the other authors’ ROC *p* values, than the original model.

Now that step eight had produced a final model that included 12 variables, we decided to test the addition of some alternative variables that were not included in the Lee et al. (2006) or Kobayashi et al. (2017) analyses to see if they improved performance. In step nine we examined the inclusion of 15 self-report variables from the TILDA survey. Only five of these variables remained significant after inclusion in a multivariable regression with four-year mortality as the outcome. Two of these five variables were subsequently dropped as they were negatively associated with mortality and we are examining positive associations. The remaining three variables (self-described physical health; ED admit OR inpatient admit in last year; and ED admit OR inpatient admit in last year OR residential care admit in last year). As there may be an overlap in the last two variables, they were assessed separately. A first multivariable regression was ran including the original model from step seven, self-described poor physical health and the first admission variable. A second multivariable regression was then ran, but it included the second admission variable instead. Based on *p* values and overlap, the second admission variable was dropped (ED admit OR inpatient admit in last year OR residential care admit in last year). This left a model of 14 variables (including the two remaining self-report variables).

In step 10, the same weighting system as step eight was applied to this updated self-report model. To validate each model, we calculated the receiver operating characteristic (ROC) curves for the updated self-report model using the validation cohort. The ROC *p* value scores were then compared between the original model from step eight and the updated self-report model from step nine. The updated self-report model performed better than the original model. This 14-variable model is our final four-year mortality index for the Republic of Ireland. The model is as follows: male; ages 65-69; ages 70-74; ages 75-79; ages 80-84; ages 85+; heart attack; cancer; smoking past age 30; difficulty with walking 100m; difficulty using the toilet; difficulty lifting 10lbs; self-reported poor physical health; and ; ED admit OR inpatient admit in last year.

**2. Variables included in analysis**

*Demographics*: This category includes biological sex and age. Biological sex was denoted as male or female, and ages in years were assigned to one of seven groupings (50-59, 60-64, 65-69, 70-74, 75-79, 80-84, 85+).

*Cardiovascular illness*: This category includes individual CV diagnoses (heart-attack, heart failure, stoke, angina, arrhythmia, cholesterol and hypertension) and grouped CV diagnoses (any serious condition (one of stroke, heart attack, heart failure or angina); and any risk factor (one of arrhythmia, cholesterol or hypertension)). A diagnosis from a doctor was required for all illness questions (yes/no). Participants were not permitted to self-diagnose.

*Non-cardiovascular diagnosis of serious illness*: This category includes diabetes, cancer, lung disease, dementia, psychological or emotional mental health problem, and arthritis. As above, a diagnosis from a doctor was required for all illness questions (yes/no). Participants were not permitted to self-diagnose.

*Health and lifestyle variables*: This category includes current smoker, smoked in past, current smoker or smoked past age of 30, pain status, daily alcohol use, falls, incontinence, visual impairment, and hearing impairment. All variables required yes/no answers. Pain status queried ‘are you often troubled by pain?’. Falls queried ‘have you fallen in the last year?’. Incontinence queried ‘during the last 12 months, have you lost any amount of urine beyond your control?’. Visual impairment queried ‘are you self-described as poor/legally blind or diagnosed with an eye disease?’. Hearing impairment queried ‘do you use a hearing aid or are you self-described as having poor hearing?’.

*Functional variables*: This category focuses on difficulties with activities of daily living. Variables include difficulties with: managing money, walking 100 meters, climbing one flight of stairs, climbing several flights of stairs, lifting heavy objects, bathing, toileting, using the telephone, getting out of bed, eating, walking across a room, dressing, shopping for groceries, taking medication, vigorous physical exercise in the last week, preparing meals, lifting 10lbs, stooping or reaching or crawling, reaching arms above shoulders, getting up from a chair, completing household chores, and sitting for long periods of time. Each question required a yes or no answer.

*Self-Report and Healthcare Utilization variables*: This category includes seven self-reported physical and mental health variables. These included: describes own physical health as poor, describes own mental health as poor, feels depressed all the time, sleep is restless all the time, feels lonely all the time, reports having a long-term health problem, illness disability or infirmity, and has difficulty falling asleep. This category includes three social interaction variables: participates in sports/social groups, has zero friends they feel close to, and has zero family they feel close to. This category includes six healthcare utilization variables: more than 50% chance of moving into nursing home in next 5yrs, more than 50% chance of developing cognitive problems aged 75+, more than 1 emergency department admission in last year, more than 1 emergency department or inpatient admission in last year, and more than 1 emergency department or inpatient or residential care admission in last year.
